# Microbiological and Mycotoxicological Quality of Stored Wheat, Wholemeal Flour and Bread: The Impact of Extreme Weather Events in Romania in the 2024 Summer

**DOI:** 10.3390/toxins17100502

**Published:** 2025-10-11

**Authors:** Valeria Gagiu, Elena Mirela Cucu (Chirtu), Elena Iulia Lazar (Banuta), Cristian Mihai Pomohaci, Alina Alexandra Dobre, Gina Pusa Pirvu, Oana Alexandra Oprea, Cristian Lazar, Elena Mateescu, Nastasia Belc

**Affiliations:** 1 National Research & Development Institute for Food Bioresources (IBA Bucharest), 020323 Bucharest, Romania; mirela.cucu@bioresurse.ro (E.M.C.); informaticaro@gmail.com (C.M.P.); alina.dobre@bioresurse.ro (A.A.D.); gina.constantinescu@bioresurse.ro (G.P.P.); nastasia.belc@bioresurse.ro (N.B.); 2 Faculty of Agriculture, University of Agronomical Sciences and Veterinary Medicine of Bucharest (USAMV Bucharest), 011464 Bucharest, Romania; 3 SC Sapte Spice SA, 240630 Ramnicu Valcea, Romania; iulia.lazar@saptespice.ro (E.I.L.); cristian.lazar@saptespice.ro (C.L.); 4 Faculty of Agricultural Sciences, Food Industry and Environmental Protection, Lucian Blaga University of Sibiu, 550012 Sibiu, Romania; 5 National Meteorological Administration (METEO-Romania), 013686 Bucharest, Romania; oprea@meteoromania.ro (O.A.O.); elena.mateescu@meteoromania.ro (E.M.)

**Keywords:** milling and baking, stored wheat contamination, wholemeal flour safety, wholemeal bread quality, *Fusarium*-damaged kernel, deoxynivalenol, aflatoxin B1, ochratoxin A, extreme drought impact, climate change

## Abstract

This study examines the effects of the extreme drought and heatwaves that occurred in Romania during the summer of 2024 on the microbiological and mycotoxicological quality of wheat (*Triticum aestivum*) stored until April 2025, as well as on the quality of wholemeal flour and bread derived from it. Comparative analyses were conducted against the contamination in wheat harvested in 2024. The hot and dry conditions significantly influenced the microbial and mycotoxicological contamination of both freshly harvested and stored wheat, as well as the derived flour and bread, due to their notably reduced moisture content and water activity. Although levels of total fungi, *Fusarium*-damaged kernels, and mycotoxins deoxynivalenol, aflatoxin B1, and ochratoxin A remained well below regulatory thresholds, higher contamination was observed in Transylvania and Moldavia—particularly in the Curvature Carpathians, likely due to their cooler and wetter microclimates. The observed quality changes were strongly associated with alterations in physico-chemical, rheological, and colorimetric parameters, posing potential economic challenges for the milling and baking industries. The study recommends implementing integrated regional strategies to enhance wheat resilience, optimize production systems, and improve contamination control in response to increasing climate stress across Southeastern Europe.

## 1. Introduction

Annually, mycotoxin contamination affects a substantial proportion—estimated at 60–80%—of the global cereal harvest, resulting in serious economic losses and health risks for both humans and animals [1,2,3]. Cereal contamination with fungi and mycotoxins begins in the field due to weather conditions, continues during transport and storage, and is influenced by milling, fermentation, and baking processes [4,5,6,7,8,9]. The mill plays a key role in grain processing because fungi and mycotoxins are concentrated in the bran, the outer layers of the grain, while refined flour is much cleaner [5,10]. Wheat processing significantly reduces mycotoxin contamination, especially through debranning (up to 70%) and milling (60–80% in white flour), and cleaning contributes 20–40% [5]. During the baking process, mycotoxins present in contaminated dough exhibit high thermal stability, which limits the efficiency of heat treatment in reducing concentrations up to 40% for deoxynivalenol, 12–30% for aflatoxin B1, and 20% for ochratoxin A [5,11,12,13]. To reduce or prevent contamination in the cereal supply chain, pre- and post-harvest strategies have been developed, along with the adoption of Good Agricultural Practices (GAP) and Hazard Analysis and Critical Control Points (HACCP) systems [4,14].

However, the most significant factor in fungal and mycotoxin contamination in cereals is annual weather conditions, especially extreme events such as heavy precipitation, floods, heatwaves, and droughts, which are intensified by climate change [8,9,15,16,17].

In 2024, global and European average temperatures reached record highs of 15.10 °C and 10.69 °C, respectively, primarily due to global warming and the El Niño phenomenon [9,18,19]. Europe experienced flooding in the northern areas and heatwaves and severe droughts in regions such as Spain, western and southeastern France, central Italy, Hungary, western and southeastern Slovakia, western and southern Romania, western Bulgaria and Greece, eastern Ukraine, and southwestern Russia [9,19,20]. Crop yields in several European Union countries were below the five-year average, with Romania, Hungary, Bulgaria, and Greece being the most severely affected [9,20].

In Romania, the 2024 wheat harvest yielded lower quantities but improved quality compared to the 2023 harvest. In contrast, corn had aflatoxin B1 contamination due to precipitation in July and August 2024, a situation also reported for Bulgaria, Serbia, and Hungary [9]. The severe drought in June greatly impacted the wheat harvest, leading the European Commission and the Romanian Government to offer compensatory payments and credit facilities to support affected farmers [21,22]. The wheat quality was better than the previous year, with a ratio of 70% bakery to 30% feed, compared to 60% bakery to 40% feed in 2023 [23]. The microbiological and mycotoxicological quality of common wheat exhibited regional differences, with higher levels observed in the wetter West Plain and Transylvania regions, and lower levels in the drier southern areas. However, contamination adversely affected the physico-chemical and sensory-colorimetric qualities of the wheat, indicating possible economic losses and the spread of deoxynivalenol and aflatoxins related to climate change, especially in the northwestern part of the country [9,24]. During storage, the weather was predominantly warm and dry, conducive to safe grain storage [24,25]. The 2024 drought severely disrupted Romania’s milling and bakery sectors, as well as the broader European market, by reducing wheat production and causing volatile raw material prices. Southeastern Romania recorded significant yield declines, while low-priced grain imports from Ukraine destabilized the domestic market [26]. However, European Commission data on cereals production and pricing does not currently indicate any marked regional price increase [27]. Consequently, industry companies had to absorb additional costs and invest in modern technologies to ensure the quality and safety of their food [28]. In this context, assessing the quality of stored cereals and derived products—through microbiological and mycotoxicological methods (e.g., high-performance liquid chromatography (HPLC), gas chromatography (GC), and enzyme-linked immunosorbent assay (ELISA)), near-infrared (NIR) spectroscopy, physico-chemical testing, and texture analysis—has become essential for ensuring food safety and security [5,29,30].

This study examines the microbiological and mycotoxicological quality of stored wheat, wholemeal flour, and bread made from it, in the context of the severe drought in summer 2024. It also compares these results with the microbiological and mycotoxicological quality of common wheat harvested in 2024. Additionally, the research explores correlations between contamination levels and the products’ physico-chemical, rheological, and sensory-colorimetric properties (specific indicators will be detailed in future publications). The findings are significant for stakeholders in the agrifood industry, trade, regulatory agencies, consumers, and scientific researchers.

## 2. Results and Discussions

### 2.1. Weather Conditions in Romania in the 2024 Summer

**Figure 1:**
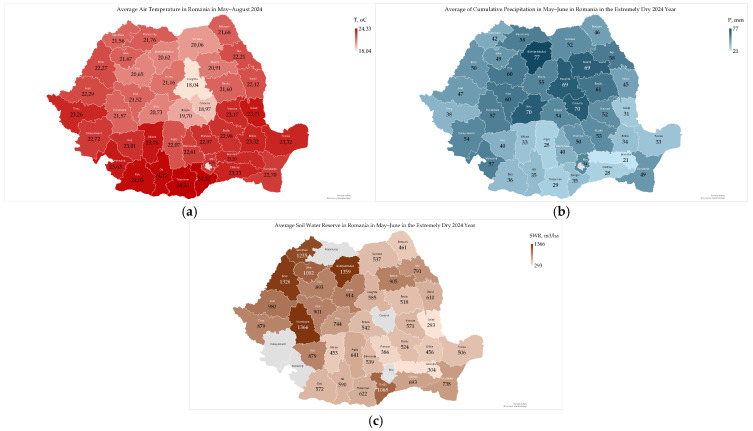
Agrometeorological conditions in Romania in the extremely dry 2024 year: (**a**) average air temperature in May–August; (**b**) average cumulative precipitation in May–August; (**c**) average soil water reserve in May–June [9,24]. There are no data available for the soil water reserve in Maramures, Covasna, Caras-Severin, Mehedinti, and Ilfov counties.

Between May and August 2024, Romania experienced unprecedented climate extremes, characterized by the highest recorded temperatures (24.33 °C, in Teleorman county) and low precipitation (21 mm, in Ialomita county) in the Southern Plain, and severe, widespread soil drought (293 m^3^/ha in Galati county) in southern Moldavia (Figure 1a–c). June, July, and August were the warmest months on record, affecting wheat and corn harvests [9,24]. The summer temperatures averaged approximately 3.5 °C above the 1991–2020 norm, and a historic 46-day heatwave prompted numerous Red Code warnings from the National Meteorological Administration [24].

These extreme heat and persistent drought conditions were consistent with broader European patterns, where Romania, situated in the Southeast, faced some of the continent’s highest temperature anomalies (+2.5 °C to +3 °C above average) and drought conditions. This contrasted with Western Europe’s significant flooding, thereby highlighting a clear climatic division across Europe during this period [9,18,19].

### 2.2. Moisture

**Figure 2:**
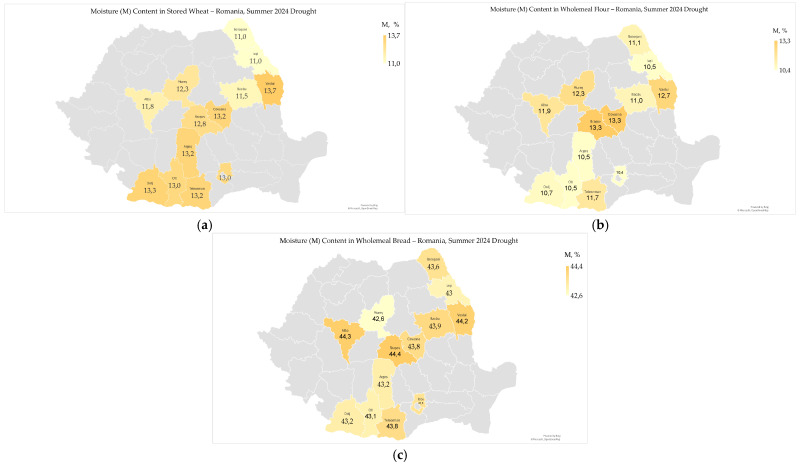
Moisture in stored wheat (**a**), wholemeal flour (**b**), and wholemeal bread (**c**), following heatwaves and extreme drought in the summer of 2024 in Romania.

Moisture content (M) ranged from 11.0% to 13.7% (12.5 ± 0.9%) in stored wheat, from 10.4% to 13.3% (11.5 ± 1.10%) in wholemeal flour, and from 42.6% to 44.5% (43.6 ± 0.5%) in wholemeal bread (Figure 2a–c).

Moisture is crucial for the microbiological and mycotoxicological safety of wheat, flour, and bread, as it affects their physico-chemical and rheological properties, as well as the baking process and the Maillard reaction that influences crust color [31,32,33]. Moisture levels above 14.5% in wheat, 14% in flour, and 40% in bread promote the growth of toxigenic fungi from the genera *Fusarium*, *Aspergillus*, and *Penicillium* [34,35,36]. To prevent fungal growth and mycotoxin production, it is important to store flour in environments with humidity below 65%, keep bread in dry places, consume it within a reasonable timeframe, and use packaging that limits moisture absorption [35].

The average moisture content of wholemeal flour was similar to that of common wheat at harvest in the extremely dry 2024 summer (11.5% versus 11.3%), due to weather conditions and environmental control in storage silos [9,24,25]. These moisture values were well below the minimum value of 14% for fungal and bacterial growth during storage of wheat and flour [36].

Analysis of the geographical distribution of moisture content in stored wheat showed that it was lower in northern counties and higher in southern counties (Figure 2a). The moisture distribution in wheat harvested in 2024 was significantly to very significantly correlated with regional agroclimatic conditions, specifically low moisture content in wheat in the hot and dry southern regions of Oltenia Plain, Southern Plain, and Dobrogea, and higher moisture content in wheat in the colder and wetter northern regions of Transylvania and Moldova (Figure 1 and Figure 2) [9,24]. The inverse distribution of moisture content in the harvested wheat and the stored wheat suggests that wheat from northern areas underwent a drying process before storage. Conversely, bread made from northern wheat had higher moisture content (Figure 1c) because flour tends to absorb more water during dough kneading and baking, which affects the texture, kneading process, and shelf life of bread [37]. This geographical distribution is similar to that of moisture content in harvested wheat from 2000 to 2014, which varied annually and regionally, and significantly influenced other harvest indicators such as hectoliter mass, Hagberg falling number, protein content, gluten content, and the incidence of *Fusarium*-damaged kernels [38].

Moisture in wholemeal flour showed a significant correlation with high temperatures from May to August (r_xy_ = −0.703 **) and precipitation in August (r_xy_ = 0.577 *); however, it did not correlate with soil water reserves in May and June 2024 (Table S1). High temperatures likely reduced flour moisture through faster grain drying, while August precipitation may have increased it by delaying harvest; early soil water reserves had a limited effect, possibly due to later rainfall. Moisture in wholemeal flour and harvested wheat decreased due to the high temperatures from May to August and increased with precipitation in August 2024 [9]. Moisture in wholemeal bread was not significantly correlated with agrometeorological parameters in 2024, which may be due to the geographic distribution and increased hydration capacity of dry flour [37].

Moisture content was correlated with total fungi in stored wheat (r_xy_ = −0.743 **), water activity (r_xy_ = 0.985 ***), and deoxynivalenol (r_xy_ = 0.791 **) in wholemeal flour, as well as ochratoxin A in wholemeal bread (r_xy_ = 0.662 *). It showed no correlation with other microbiological and mycotoxicological indicators (Table S2). In common wheat 2024, moisture was correlated with water activity across all regions (r_xy_ = 0.416 * to r_xy_ = 0.971 ***), and with deoxynivalenol in the Southern Plain (r_xy_ = −0.596 **); however, it did not correlate with total fungi, *Fusarium*-damaged kernels, or total aflatoxins in any region [9].

Moisture showed significant correlations with the physico-chemical and rheological properties of wholemeal flour and bread (Table S3). In wholemeal flour, moisture was highly significantly correlated with total titratable acidity (r_xy_ = 0.752 ***) and starch content (r_xy_ = 0.920 ***). Similarly, in wholemeal bread, moisture was highly significantly correlated with total titratable acidity (r_xy_ = 0.874 ***), protein content (r_xy_ = 0.894 ***), fat (r_xy_ = 0.840 ***), and ash (r_xy_ = 0.910 ***) (Table S3). These correlations reflect the direct influence of soil and climate conditions on the biochemical composition of the products, particularly during an extremely dry year in 2024, when water stress resulted in higher concentrations of solids in wheat grains [39,40]. In 2024, moisture content was significantly correlated with protein and wet gluten in harvested wheat in Oltenia Plain (r_xy_ = −0.679 ***, and r_xy_ = −0.649 ***) and Dobrogea (r_xy_ = −0.856 ***, and r_xy_ = −0.761 ***) regions, which were the most affected by high temperatures and extreme soil drought [9]. The chemical composition, fluidity, storage, and shelf life of wheat flour are influenced by moisture content and water activity [41,42].

Moisture correlated with sensory-colorimetric indicators only in wholemeal flour (L* − whiteness, r_xy_ = 0.592 *; a*–redness, r_xy_ = −0.624 *; and b*–yellowness, r_xy_ = −0.665 *) (Table S4). In 2024, the moisture content and sensory-colorimetric indicators of common wheat showed a negative and very significant correlation in the Oltenia Plain and Dobrogea regions [9]. This indicates that the heatwave and extreme drought in the 2024 summer negatively affected the moisture content in common wheat and, implicitly, the sensory-colorimetric properties of wholemeal flour [9,43].

The moisture content of wholemeal flour showed noticeable variations, mainly due to the high temperatures from May to August and the heavy precipitation in August 2024. These weather conditions significantly affected the microbiological and mycotoxicological safety, as well as the physico-chemical, rheological, and sensory-colorimetric qualities of the wheat, flour, and bread [9]. The trend of wheat moisture in Romania, in the context of climate change, reveals significant yearly and regional differences. These variations are driven by weather patterns, with higher moisture levels recorded in the colder, wetter areas of Transylvania and northern Moldavia, and lower moisture levels in the warmer, drier southern regions [38].

### 2.3. Water Activity

**Figure 3:**
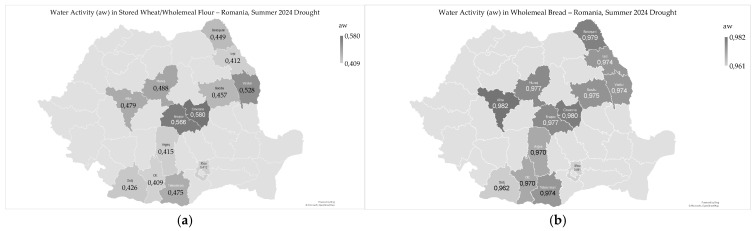
Water activity in stored wheat/wholemeal flour (**a**) and wholemeal bread (**b**), following heatwaves and extreme drought in the summer of 2024 in Romania.

Water activity (aw) ranged from 0.409 to 0.580 (0.469 ± 0.059) in stored wheat/wholemeal flour and from 0.961 to 0.982 (0.973 ± 0.006) in wholemeal bread (Figure 3a,b).

Water activity is crucial for food stability and quality, as high values favor microbial growth and enzymatic reactions in flour and bread, leading to food spoilage. The optimal air temperature and water activity values for fungal growth and mycotoxin production during grain storage are as follows: *Fusarium* spp./Deoxynivalenol, temperature 20–25/29–30 °C and aw 0.98–0.99/0.99; *Aspergillus flavus*/Aflatoxin, temperature 35/28 °C and aw 0.95/0.99; and *A. ochraceus*/Ochratoxin A, 30/25–30 °C and aw 0.96–0.98/0.98 [44]. Water activity during wheat storage is influenced by in-field and post-harvest values: aw 1.00 is favorable for the development of *Fusarium* spp. in the field, and aw <0.95 favors the growth of *Aspergillus* spp. and *Penicillium* spp. in the post-harvest phase [9,44].

The average water activity in wholemeal flour is similar to that in common wheat at harvest in the extremely dry 2024 year (aw 0.469 versus 0.463), due to weather conditions and environmental control in storage silos [9,24,25]. Low water activity values (aw < 0.60) in flour prevent microbial growth; however, spores and dormant microorganisms that remain viable pose a potential health risk [45]. The minimum water activity values for fungal growth and mycotoxin production are as follows: *Fusarium* spp./Deoxynivalenol, aw 0.90–0.91/0.90; *A. flavus*/Aflatoxin, aw 0.78–0.84/0.84; and *A. ochraceus*/Ochratoxin A, aw 0.77/0.83–0.87 [44].

The analysis of the geographical distribution of water activity in wheat products revealed higher values in the intra-Carpathian and northeastern counties of Romania, both for stored wheat/wholemeal flour, as well as for wholemeal bread (Figure 3a,b). In 2024, water activity in harvested wheat had higher values in wheat grown on acidic soils of the Carpathian areas [9]. The lowest water activity values in stored wheat, wholemeal flour, and bread were recorded in the south of the country, which experienced extreme drought in 2024 (Figure 3a,b) [9].

Water activity in wholemeal flour and bread correlated significantly with high temperatures from May to August (flour, r_xy_ = −0.740 **; and bread, r_xy_ = −0.641 *) and with precipitation in August (flour, r_xy_ = 0.608 *; and bread, r_xy_ = 0.631 *), but not with soil water reserves in May and June 2024 (Table S1). Water activity in stored wheat, wholemeal flour, and bread was reduced by high temperatures from May to August and increased by precipitation at harvest in 2024.

Water activity in wholemeal flour correlated very significantly with moisture content (r_xy_ = 0.985 ***) and deoxynivalenol (r_xy_ = 0.805 ***), but did not correlate with other microbiological and mycotoxicological indicators; no correlations were observed in wholemeal bread (Table S2). In 2024, water activity in harvested wheat correlated significantly only with deoxynivalenol in Dobrogea; it did not correlate with total fungi, *Fusarium*-damaged kernel, and total aflatoxins in any region [9].

Water activity showed significant correlations with the physico-chemical and rheological properties of wholemeal flour and bread (Table S3). In wholemeal flour, water activity was very significantly correlated with moisture (r_xy_ = 0.985 ***), total titratable acidity (r_xy_ = 0.719 ***), and starch content (r_xy_ = 0.861 ***). Similarly, in wholemeal bread, water activity was very significantly correlated with moisture (r_xy_ = 0.998 ***), total titratable acidity (r_xy_ = 0.875 ***), protein content (r_xy_ = 0.887 ***), fat (r_xy_ = 0.837 ***), and ash (r_xy_ = 0.901 ***) (Table S3). These correlations highlight the impact of drought-driven soil and climate conditions, which led to a higher concentration of solids in wheat due to water stress [39,40]. In 2024, a positive correlation was observed between water activity and protein content in harvested wheat in the western part of the country, while a negative correlation was found in the southwest, which was attributed to extreme drought in June [9]. The chemical composition, fluidity, storage, and shelf life of wheat flour are influenced by water activity [41,42].

Water activity correlated significantly with sensory-colorimetric indicators only in wholemeal flour (a*–redness, r_xy_ = −0.580 *; and b*–yellowness, r_xy_ = −0.618 *) (Table S4). In 2024, a negative correlation was observed between water activity and sensory-colorimetric indicators in the southern part of the country, attributed to plant stress resulting from extreme drought [9]. This suggests that the extreme drought in 2024 had a negative impact on water activity and, consequently, the sensory properties of wholemeal flour [9,43].

Water activity in wholemeal flour was significantly influenced by moisture content, affecting deoxynivalenol contamination, with higher values observed in the intra-Carpathian and northeastern regions of Romania. Also, water activity showed a significant correlation with traditional flour and bread quality metrics. This trend was consistent in 2024, highlighting the significant impact of environmental conditions on moisture content and water activity in grains, as well as on food quality [9,38].

### 2.4. Total Fungi

**Figure 4:**
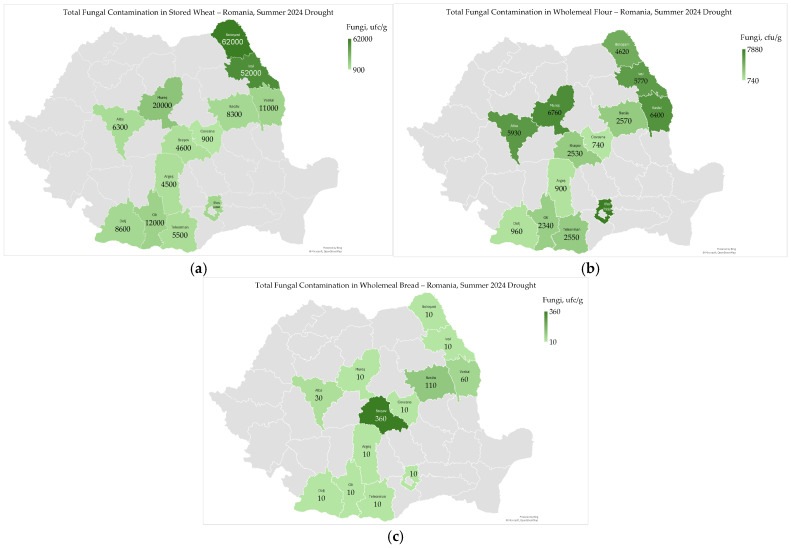
Total fungi in stored wheat (**a**), wholemeal flour (**b**), and wholemeal bread (**c**), following heatwaves and extreme drought in the summer of 2024 in Romania.

Total fungal contamination ranged from 900 cfu/g to 62,000 cfu/g (mean 15,823 cfu/g) in stored wheat, from 740 cfu/g to 7880 cfu/g (mean 3842 cfu/g) in wholemeal flour, and from <10 cfu/g to 360 cfu/g (mean 140 cfu/g) in wholemeal bread (Figure 4a–c).

Effective agricultural practices and proper storage management are crucial for minimizing fungal growth and mycotoxin risks in raw wheat, as high fungal counts indicate potential spoilage and food safety concerns [46]. Processing steps such as milling and baking significantly reduce fungal contamination, resulting in very low fungal counts in bread, which is crucial for its safety and shelf life [47,48]. However, mycotoxins may persist despite baking, underscoring the importance of monitoring fungal contamination in raw materials to mitigate these risks [49]. According to Order No. 27/2011 of the National Veterinary Sanitary and Food Safety Authority, the maximum allowed contamination with total fungi varies as follows: from 10,000 to 100,000 cfu/g in consumer wheat, at the end of the technological process; from 100 to 10,000 cfu/g for flour varieties with an extraction degree higher than 650 (which also includes wholemeal flour); from 10 to 1000 cfu/g for bakery flours and flours with a low extraction degree (types 480, 550 and 650) and from 10 to 100 cfu/g in bread [50].

The fungal contamination in the wheat chain significantly decreased from stored wheat through wholemeal flour to wholemeal bread (Figure 4a–c). Total fungal contamination in stored wheat and wholemeal flour was higher in the northern half of the country (Figure 4), as the moisture content of wheat is higher in the Transylvania and Moldavia regions due to colder and wetter climate [38]. Fungal contamination had a similar geographic distribution to that of moisture content and water activity in wheat harvested in 2024 and stored wheat (Figure 2, Figure 3 and Figure 4) [9].

Fungal contamination and agro-meteorological conditions were significantly correlated (Table S1). In June 2024, fungal contamination in wholemeal flour was significantly correlated with low precipitation and soil water reserves (r_xy_ = 0.563 *; and r_xy_ = 0.779 **). Stored wheat did not show these correlations, meaning that wholemeal flour is more susceptible to contamination due to its higher bran content. This is due to the dry milling process, which redistributes contamination across flour fractions [45]. In 2024, fungal contamination was correlated with drought conditions in the southern part of the country, specifically in the Oltenia Plain and Dobrogea regions [9]. Drought conditions lead to changes in the structure of soil fungal colonies, which also impact wheat contamination [8,16,51].

Total fungal contamination was correlated only with moisture content in wheat (r_xy_ = −0.743 **), but not with *Fusarium*-damaged kernels and the mycotoxins deoxynivalenol, aflatoxin B1, and ochratoxin A (Table S2). However, moisture content itself was positively correlated with water activity, deoxynivalenol, and ochratoxin A (Table S2). This suggests that while fungal biomass may increase under dry conditions, mycotoxin production is more sensitive to moisture availability, particularly during grain development and storage [52].

Total fungal contamination was not significantly correlated with physico-chemical, rheological, and sensory-colorimetric quality indicators of stored wheat, wholemeal flour, and bread (Tables S3 and S4). This means that the presence of fungi in wheat may increase under drought conditions, leading to potential microbiological contamination despite acceptable physico-chemical properties [9].

Fungal contamination in stored wheat is significantly reduced through milling and baking; however, moisture content, dry conditions in the summer of 2024, and storage practices influenced contamination levels and mycotoxin persistence [46,47]. Still, mycotoxins persisted despite baking, underscoring the importance of monitoring fungal contamination in raw materials to mitigate these risks [49]. Proper agricultural and storage methods are crucial for mitigating risks, especially in the context of climate change [9].

### 2.5. Fusarium-Damaged Kernel

**Figure 5:**
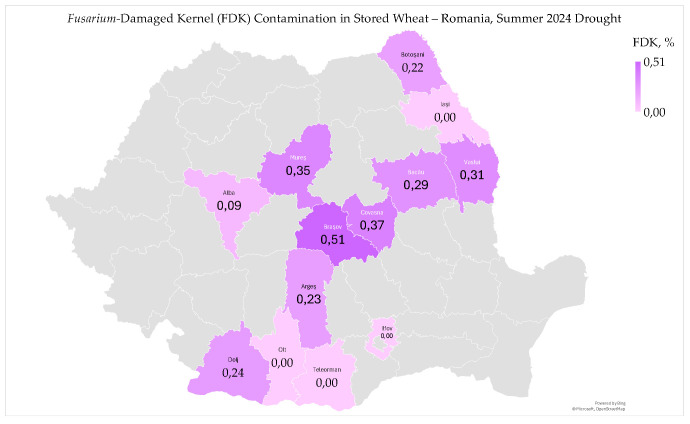
*Fusarium*-damaged kernel contamination in stored wheat, following heatwaves and extreme drought in the summer of 2024 in Romania.

*Fusarium*-damaged kernel (FDK) ranged from 0% to 0.51% (0.20 ± 0.17%) in stored wheat (Figure 5).

*Fusarium graminearum*, a plant pathogen, thrives in temperate, warm, and subtropical regions with moderate to high precipitation, which aligns with major wheat-producing areas [53]. In Romania, *Fusarium* contamination in wheat is closely correlated with heavy precipitation, especially in the northwest and west, and has a significant impact on crop quality [8,9]. *Fusarium* infection degrades the quality of wheat through mycotoxin contamination, resulting in financial losses for farmers, trade disruptions, and a significant risk to food safety, affecting both processors and end consumers [8,9,54].

*Fusarium*-damaged kernel contamination in stored wheat was classified as good or very good, according to the National Grading Plan (Figure 5) [9,55]. In 2024, common wheat was also rated as good or very good, with FDK contamination exceeding the 1% limit in only two samples from West Plain and Transylvania, specifically in Bihor and Mures counties [9]. The FDK contamination levels were lower in the regions of Dobrogea, Southern Plain, Moldavia, and Oltenia Plain, and higher in the West Plain, Southern Hilly Area, and Transylvania [9].

*Fusarium*-damaged kernel contamination in stored wheat significantly correlated with high temperatures from May to August 2024 (r_xy_ = −0.729 **); still, it did not correlate with precipitation and soil water reserve in May to June (Table S1). FDK contamination in common wheat in 2024 significantly correlated with precipitation and soil water reserve in May and June [9].

*Fusarium*-damaged kernel contamination was not significantly correlated with other microbiological and mycotoxicological indicators (total fungi, deoxynivalenol, aflatoxin B1, and ochratoxin A) in stored wheat (Table S2).

*Fusarium*-damaged kernel contamination was correlated only with hectoliter mass (r_xy_ = −0.629 *) and wet gluten content (r_xy_ = −0.642 *), but not with other physico-chemical and rheological indicators in stored wheat (Table S3). In common wheat harvested in 2024, FDK contamination showed a significant correlation with total fungi, the mycotoxins deoxynivalenol and total aflatoxins, as well as with physico-chemical and sensory-colorimetric indicators. These correlations exhibited notable variations across different agricultural regions [9].

The study on *Fusarium*-damaged kernel contamination in common wheat harvested in 2024 and stored wheat reveals its negative impact on wheat quality, and the need for improved agricultural practices and comprehensive testing methods to ensure food safety. It highlights the economic implications due to decreased market value in years with extreme weather events and suggests specific interventions in regions prone to contamination [54,56]. Climate change is expected to increase the severity of *Fusarium* Head Blight (FHB) in wheat, and the mycotoxins produced will pose a threat to both the quality and safety of grain and human health [57].

### 2.6. Deoxynivalenol

**Figure 6:**
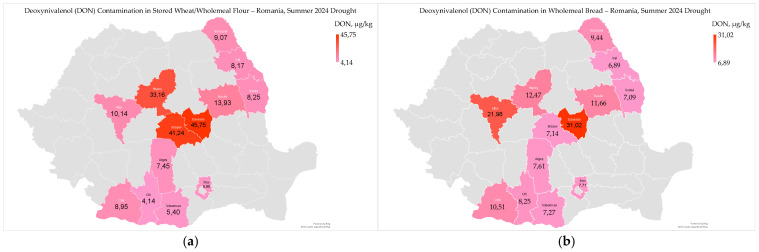
Deoxynivalenol contamination in stored wheat/wholemeal flour (**a**) and wholemeal bread (**b**), following heatwaves and extreme drought in the summer of 2024 in Romania.

Deoxynivalenol (DON) ranged from 4.14 µg/kg to 45.75 µg/kg (15.50 ± 14.43 µg/kg) in stored wheat/wholemeal flour, and from 6.89 µg/kg to 31.02 µg/kg (11.46 ± 7.16 µg/kg) in wholemeal bread (Figure 6a,b).

Deoxynivalenol (also known as vomitoxin) is a mycotoxin produced by fungi of the genus *Fusarium*, which often contaminates cereals such as wheat, maize, and barley [58]. Deoxynivalenol is not classified as carcinogenic to humans (Group 3). However, it is a toxic substance that can harm health by causing nausea, vomiting, diarrhea, and abdominal pain if ingested in large amounts or over an extended period [59,60]. The maximum allowable levels of deoxynivalenol are 1000 µg/kg in unprocessed cereal grains, 600 µg/kg in cereal milling products, 600 µg/kg in pasta, 400 µg/kg in baked goods, cereal bars, and breakfast cereals, and 150 µg/kg in foods for special medical purposes for infants and young children [61]. The European Food Safety Authority (EFSA) has set a tolerable daily intake (TDI) of 1 µg/kg of body weight for deoxynivalenol [61].

Deoxynivalenol contamination in stored wheat/wholemeal flour and wholemeal bread was well below the limits in unprocessed wheat, milled products, and bakery goods [61]. Contamination levels were higher in Covasna, Brasov, and Mures counties, which are situated within the Curvature Carpathians and have a cooler, more humid climate (Figure 6a,b). In 2024, deoxynivalenol levels in harvested wheat were higher in western and southwestern Romania but remained below the maximum permitted limit [9].

Deoxynivalenol contamination in wholemeal flour and bread correlated with high temperatures from May to August (flour, r_xy_ = −0.843 ***; and bread, r_xy_ = −0.571 *) and precipitation in August (flour, r_xy_ = 0.580 *; and bread, r_xy_ = 0.616 *), but not with soil water reserve in May and June 2024 (Table S1). This indicates that precipitation during wheat harvest increases the likelihood of contamination with deoxynivalenol, and the mycotoxin is subsequently found in stored wheat, wholemeal flour, and bread [9,62].

Deoxynivalenol contamination was significantly correlated only with moisture content and water activity (r_xy_ = 0.791 **; and r_xy_ = 0.805 ***) in wholemeal flour; no correlations were found with other microbiological and mycotoxicological indicators in flour and bread (Table S2). High moisture content and water activity are the primary factors contributing to deoxynivalenol contamination in wholemeal flour, as they promote the growth of *Fusarium* fungi. Controlling moisture levels during storage and processing is crucial to mitigate mycotoxin risks [52].

Deoxynivalenol contamination did not correlate with physico-chemical indicators, but only with dough rheological indicators (maximum pressure resistance of the dough, r_xy_ = −0.724 **; and P/L ratio, r_xy_ = −0.622 *) (Table S3) and sensory-colorimetric indicators (a* − redness, r_xy_ = −0.662 *) in wholemeal flour (Table S4). This indicates that deoxynivalenol significantly reduces the quality of wholemeal flour, particularly in terms of baking properties (dough strength and elasticity) and color [63,64].

Deoxynivalenol contamination levels in stored wheat/wholemeal flour and bread were significantly lower than the established regulatory limits. Higher contamination was observed in the intra-Carpathian counties, suggesting that environmental factors play a significant role. This geographical location was also identified as a common wheat hotspot in the extremely dry year 2015, from June to August [6,8]. No correlation was found between deoxynivalenol contamination and other microbiological, mycotoxicological, and physico-chemical and rheological indicators (except for moisture, water activity, dough strength, and elasticity), indicating a complex relationship with environmental conditions.

### 2.7. Aflatoxin B1

**Figure 7:**
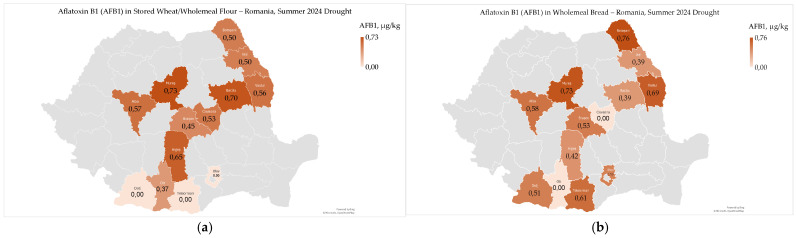
Aflatoxin B1 contamination in stored wheat/wholemeal flour (**a**) and wholemeal bread (**b**), following heatwaves and extreme drought in the summer of 2024 in Romania.

Aflatoxin B1 (AFB1) ranged from 0 µg/kg to 0.73 µg/kg (0.43 ± 0.26 µg/kg) in stored wheat/wholemeal flour and from 0 µg/kg to 0.76 µg/kg (0.47 ± 0.24 µg/kg) in wholemeal bread (Figure 7a,b).

Aflatoxin B1 is produced by the fungi *Aspergillus flavus* and *A. parasiticus*; it is highly toxic and classified by the IARC as Group 1, indicating that it is carcinogenic to humans [59,60]. Contamination with aflatoxin B1 is more likely in warm and humid environments, especially at temperatures above 25 °C and high relative humidity, which promote the growth of *Aspergillus* fungi in improperly stored cereals and foods [65,66]. The European Commission has established a maximum permissible limit of 2 μg/kg for aflatoxin B1 and 4 μg/kg for total aflatoxins in all cereals and cereal products, including processed cereal products [9,67]. Since aflatoxin B1 is genotoxic and carcinogenic, there is no set tolerable daily intake, and exposure should be minimized as much as possible [65].

Aflatoxin B1 contamination in stored wheat, wholemeal flour, and bread was well below the maximum permitted limit of 2.0 μg/kg; it showed uneven distribution across different regions, both within and outside the Carpathian area (Figure 7a,b) [67]. In 2024, harvested wheat also displayed uneven levels of total aflatoxin contamination, with the highest concentrations in the northwest of the country [9]. The varied geographical distribution and diverse agroclimatic conditions in these agricultural regions highlight the ability of fungi in the genus *Aspergillus* to grow and produce total aflatoxins, including aflatoxin B1, under different environmental conditions in Romania and Europe [7,9,15,66].

Aflatoxin B1 contamination in wholemeal flour showed a significant correlation with high temperatures from May to August (r_xy_ = −0.625 *), with the correlation being stronger in extremely dry June (r_xy_ = −0.684 **). Additionally, there was a significant correlation with August precipitation (r_xy_ = 0.654 *). However, no correlation was found with soil water reserves in May and June 2024 (Table S1) [24]. In 2024, total aflatoxin contamination in harvested wheat significantly correlated with precipitation deficit in the West Plain in May 2024 and during the 2023–2024 agricultural year [9,24]. Differences between total aflatoxins in harvested wheat in 2024 and aflatoxin B1 in stored wheat and wholemeal flour may be influenced by environmental conditions in the field, post-harvest processing and storage conditions, as well as fungal interactions [9,66,68,69].

Aflatoxin B1 contamination did not show a significant correlation with microbiological, mycotoxicological, and sensory-colorimetric indicators (Tables S2 and S4). However, it was significantly correlated only with the gluten index (r_xy_ = −0.609 *) among the physico-chemical and rheological indicators (Table S3). This indicates that AFB1 contamination reduces the quality and strength of gluten, impacting the final quality of bakery products. In wheat harvested in 2024, total aflatoxin contamination was significantly correlated with *Fusarium*–damaged kernels and deoxynivalenol only in the West Plain; there were no significant correlations with physico-chemical and sensory-colorimetric indicators in any agricultural region [9].

Aflatoxin B1 contamination levels in stored wheat, wholemeal flour, and bread remained well below the maximum permissible limit, showing geographical variability and significant links to extreme weather events in the summer of 2024. Additionally, the processing of wheat into wholemeal flour and bread can affect the distribution of aflatoxins, highlighting the importance of monitoring contamination throughout the entire production process. Although aflatoxin B1 contamination is lower in wheat compared to corn, it is expected to rise significantly due to climate change [15,66,70,71].

### 2.8. Ochratoxin A

**Figure 8:**
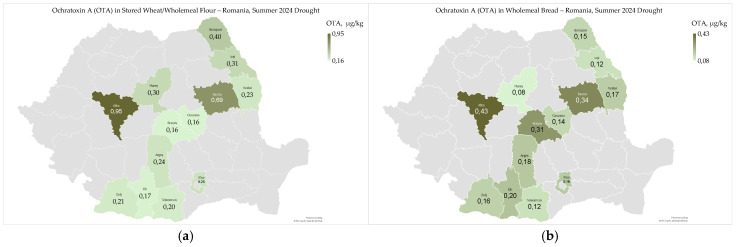
Ochratoxin A contamination in stored wheat/wholemeal flour (**a**) and wholemeal bread (**b**), following heatwaves and extreme drought in the summer of 2024 in Romania.

Ochratoxin A (OTA) ranged from 0.16 µg/kg to 0.95 µg/kg (0.33 ± 0.23 µg/kg) in stored wheat/wholemeal flour, and from 0.08 µg/kg to 0.43 µg/kg (0.20 ± 0.10 µg/kg) in wholemeal bread (Figure 8a,b).

Ochratoxin A is a mycotoxin produced by fungi of the genera *Aspergillus* (*A. ochraceus* and *A. niger*) and *Penicillium* (*P. verrucosum*), and it is considered possibly carcinogenic to humans (IARC Group 2B) due to its nephrotoxic and genotoxic effects [59,60,72]. It grows in conditions of high humidity and moderate temperatures, especially in improperly stored cereals [73]. The optimal conditions for fungal growth and mycotoxin production during grain storage are as follows: *A. ochraceus*/OTA, temperature 30/25–30 °C and aw 0.96–0.98/0.98; *P. verrucosum*/OTA, temperature −/25 °C and aw 0.95/0.90–0.95 [44]. In the European Union, the maximum permitted limits are 5 µg/kg for unprocessed cereals, 3 µg/kg for milling products, and 2 µg/kg for bakery products, and EFSA has established a tolerable daily intake of 0.4 µg/kg of body weight [73].

Ochratoxin A contamination in stored wheat, wholemeal flour, and bread was well below the maximum permitted limits; it showed a broader geographical distribution in the intra- and sub-Carpathian areas, particularly in Alba and Bacau counties (Figure 8a,b). In 2024, ochratoxin A was not analyzed in harvested wheat [9]. However, we believe that OTA production was suppressed by the very low water activity values in harvested wheat in 2024 (aw 0.595, maximum) and stored wheat/wholemeal flour (aw 0.580, maximum), We agree.likely due to heatwaves and extreme drought [9,24]. The minimum water activity required for fungal growth and ochratoxin A production in stored grains is as follows: *A. ochraceus*/OTA, aw 0.77/0.83–0.87; and *P. verucosum*/OTA, aw 0.80–0.81/0.83–0.85 [44].

Ochratoxin A contamination in stored wheat, wholemeal flour, and bread did not show a significant correlation with high temperatures from May to August, precipitation in August, or soil water reserves in May and June 2024 (Table S1) [24].

Ochratoxin A contamination did not show a significant correlation with microbiological, mycotoxicological, physico-chemical, rheological, and sensory-colorimetric indicators of stored wheat, wholemeal flour, and bread (Tables S2–S4). These results are attributed to the dry conditions at harvest and the proper storage and processing conditions of wheat and derived products (Tables S2–S4) [74].

Ochratoxin A contamination in stored wheat, wholemeal flour, and bread was within the permissible limit. This contamination was most common in the Carpathian counties, despite the extremely dry 2023–2024 agricultural year. The low water activity in harvested and stored wheat effectively prevented fungal growth and the production of ochratoxin A. No significant correlation was found between OTA levels and the quality indicators of flour and bread, or between OTA levels and weather conditions during wheat growth, storage, and processing. A study conducted in Romania from 2012 to 2015 showed the highest OTA levels in processed cereals (ensilaged corn grains and germs) in the dry southern regions, which were affected by varying precipitation levels [7].

### 2.9. Study Limitations, Future Research Directions, and Measures on Prevention and Control of Wheat Contamination in the Context of Climate Change

The study is limited by the relatively small sample size and its uneven national geographic distribution, which may affect representativeness and generalizability. However, the study benefits from the expertise gained through previous research, which strengthens its methodological approach and contextual relevance [6,7,8,9,16,38,54,66]. Based on this study, the following research directions can be developed: (1) evaluation of contamination of grains and milling and bakery products in years with successive extreme weather events (heatwave, drought, precipitation, and floods); (2) evaluation of the efficiency of different processing technologies of grains to optimize milling technologies; (3) evaluation of correlations between agricultural, technological, and commercial quality indicators of grains; (4) investigation of the long-term impact of climate change on the cereal agri-food sector.

In response to the growing frequency of extreme weather events across Romania and Europe, it is imperative to develop and cultivate wheat varieties with enhanced resilience to climatic stressors, implement efficient irrigation systems, and synchronize agrotechnical interventions to stabilize and maintain high-quality agricultural output. These efforts must be complemented by the modernization of grain milling and baking technologies, alongside rigorous monitoring of contamination risks throughout the grain supply chain, to ensure the safeguarding of food safety and the integrity of national and cross-border trade [9,17,75]. In Romania, the indigenous common wheat varieties Glosa, Izvor, Litera, Delabrad, and Miranda—developed by the National Agricultural Research and Development Institute (INCDA) Fundulea—are intensively cultivated due to their resistance to drought. Among the wheat varieties registered in European breeding programs that have demonstrated agronomic stability under drought-prone conditions, we mention the varieties SU Tarroca and Artimus–Saaten Union, Sulingen-Treben, Germany; Renan–Institut National de la Recherche Agronomique (INRAE), Paris, France; Alegoria, Opoka, Essa, Formacja–DANKO Hodowla Roślin, Choryń, Poland; and IS Spirella–Agroyoumis Sp. z o.o., Poznan, Poland. Major milling equipment manufacturers, including Bühler Group–Uzwil, Switzerland; Ag Growth International–Winnipeg, Canada; Henry Simon–Manchester, United Kingdom (currently operated in partnership with Alapala–Istanbul, Turkey, and Satake Corporation–Hiroshima, Japan); Alapala–Çorum, Turkey; and Sangati Berga–Serrana, Brazil, have modernized milling technologies to improve cleaning, optical sorting, and debranning of wheat grains, thus reducing contamination with fungi and mycotoxins [54]. These measures to prevent and reduce the contamination of cereals and cereal-based foods are particularly beneficial in national and cross-border trade, which falls under the European Commission’s Rapid Alert System for Food and Feed (RASFF).

Research must take an integrated approach, “agriculture–storage–milling–baking–food safety and security”, to determine the effects of climate change on the entire agri-food chain.

## 3. Conclusions

The study highlights the significant impact of the heatwaves and extreme drought recorded in Romania during the summer of 2024 on the microbiological and mycotoxicological quality of harvested and stored wheat, wholemeal flour, and the bread derived from it.

These extreme weather events resulted in very low values of moisture content and water activity in harvested wheat, stored wheat, and wholemeal flour, thereby affecting their microbiological and mycotoxicological quality. Although contamination levels with total fungi, *Fusarium*-damaged kernels, and the mycotoxins deoxynivalenol, aflatoxin B1, and ochratoxin A remained well below the maximum permitted limits, they exhibited higher values in the northern regions of Transylvania and Moldavia, particularly in the Curvature Carpathians. The cooler and wetter agroclimatic conditions of these regions allow contamination of cereals even in hot and dry periods. The significant correlations between microbiological and mycotoxicological indicators and physico-chemical, rheological, and sensory-colorimetric indicators of harvested wheat, stored wheat, wholemeal flour, and bread indicate a potential negative economic impact on the milling and baking industry.

The information is particularly important for Romania, Hungary, Bulgaria, and Greece, which were the most severely affected by the 2024 drought. It highlights the need for coordinated action among Southeastern European stakeholders to address drought and climate risks through the development of resilient wheat, improved farming practices, efficient irrigation systems, advanced milling and baking technologies, and ongoing monitoring of contamination to safeguard food quality and economic stability.

The upcoming publications will present the physico-chemical, rheological, and sensory-colorimetric quality of common wheat harvested in 2024, as well as stored wheat, wholemeal flour, and bread made from it, under the influence of extreme weather events in Romania during the exceptionally dry 2023–2024 agricultural year.

## 4. Materials and Methods

Our research on the microbiological and mycotoxicological quality of stored wheat, wholemeal flour, and wholemeal bread builds on our previous work on common wheat harvested in 2024, which employed a similar and complementary approach [9]. These studies assess wheat contamination under drought and high-temperature conditions from the 2023–2024 harvest through storage until April 2025. They include flour and bread derived from wheat, comparing results to trace drought effects and exploring correlations with physico-chemical, rheological, and sensory-colorimetric properties.

This integrated framework offers a model for future research initiatives.

### 4.1. Sampling and Sample Preparation

**Figure 9:**
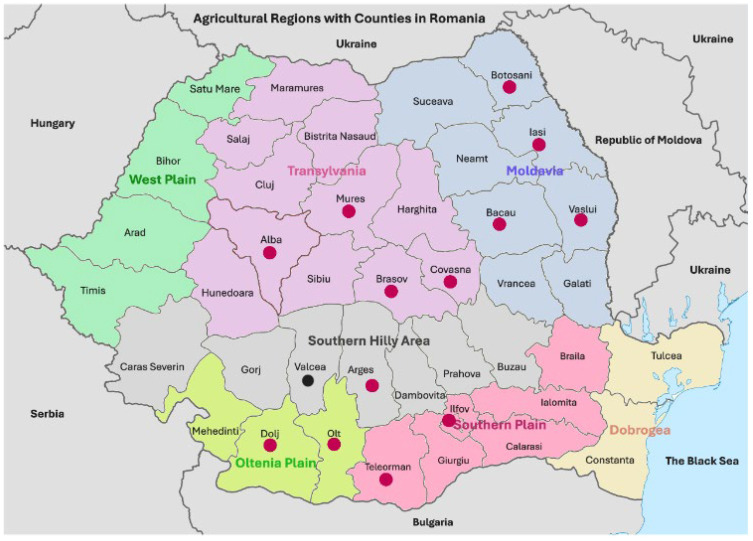
Counties from which stored wheat was purchased (red points) by the Valcea mill (black point) of the company S.C. Sapte Spice S.A.

The common wheat is from the 2024 harvest, and the samples originate from silos in thirteen counties located between 44–48 °N and 24–27 °E (Figure 9). Information on storage conditions after harvest is not available. Wheat was sampled following the SR EN ISO 24333:2010 standard [76] from batches purchased for the mill in Valcea County by the industrial milling company S.C. Sapte Spice S.A. (Figure 9) [23]. This wheat processing plant is equipped with automated Bühler machinery for cleaning, optical sorting, debranning, and a grinding line with a daily capacity of 350 tons. It also has flour silos, automatic bag packing, and mixing lines. The facility holds quality accreditation and certification in accordance with ISO standards. In 2024, the company was ranked as the top among large enterprises for manufacturing milling products.

The stored wheat was sampled once in April 2025, for the following reasons: (a) 98.8% of the 2024 harvest had moisture levels below 14%, including 85.4% classified as very dry (<12%); (b) fungal and mycotoxin contamination in the wheat harvest was very low; (c) the company applies strict wheat purchasing controls; and (d) the weather was warm and dry during the storage months [9,24,25]. Moreover, the study did not aim to investigate the temporal evolution of contamination during storage, but rather to examine the effects of the exceptionally hot and dry summer of 2024 on the microbiological and mycotoxicological quality, in conjunction with the physicochemical, rheological, and colorimetric properties of wheat, flour, and bread. 

Wholemeal flour was selected to preserve its native composition and retain the contamination profile of the stored grain, given that industrial processing and baking are known to reduce microbiological and mycotoxicological loads. To ensure analytical accuracy, all procedures were conducted under controlled laboratory conditions, deliberately excluding the Bühler processing line. 

A total of 39 samples were analyzed, including 13 stored wheat samples, 13 wholemeal flour samples, and 13 wholemeal bread samples. Each wheat sample weighed 10 kg, with 3 kg kept as grains for laboratory analysis and 7 kg ground into wholemeal flour and baked into wholemeal bread.

Grinding for laboratory analysis. Each wheat sample was sieved to remove impurities, homogenized, and ground. Common wheat was processed using a Y18 roller mill (Yücebaş Makine, Izmir, Turkey) for rheological, microbiological, and mycotoxicological analyses, and a Laboratory Mill 120 hammer mill (Perten-Perkin Elmer, Stockholm, Sweden) for gluten analyses. After grinding, the fractions were homogenized to produce wholemeal flour.

Baking test. Wholemeal flour was blended thoroughly, and the ingredients were measured and kneaded for 10 min; no enzymes or preservatives were added during the process. The dough was then removed from the mixer, divided, and pre-shaped before resting for 20 min. After resting, it was shaped, placed on trays, and proofed for 60 min. Once proofing was finished, the dough was scored and baked in an oven at 250 °C for 15 min (Classic Pro Industrial Hell Pan Oven, Craiova, Romania).

### 4.2. Microbiological and Mycotoxicological Analyses

Wheat samples were analyzed using methods accredited by the Romanian Accreditation Association (RENAR) according to the standard SR EN ISO/IEC 17025:2018 [77].

Moisture content (M, %) was determined according to the standard SR EN ISO 712:2010 [78] and using an MRC DK-500 WT laboratory oven (MRC Ltd., Holon, Israel).

Water activity (aw) was determined according to the Aquaspector AQS 31 procedure and using an Aquaspector AQS 31 (Nagy Messsysteme GmbH, Gäufelden, Germany).

Total fungi (cfu/g) were determined according to the standard SR ISO 21527-2:2009 [79] and using a Panasonic MIR-154-PE thermostat with forced cooling and ventilation at 25 °C (PHC Europe B.V., Breda, The Netherlands).

*Fusarium*-damaged kernel (FDK, %) was determined according to the standard SR EN ISO 7970:2021 [80] and using a visual method.

Deoxynivalenol (DON, µg/kg) was determined following the Ridascreen^®^ DON procedure (R-Biopharm, Darmstadt, Germany) and using a Sunrise™ plate reader at 450 nm (Tecan Group Ltd., Männedorf, Switzerland).

Aflatoxin B1 (AFB1, µg/kg) was determined following the Ridascreen^®^ Aflatoxin B1 30/15 procedure (R-Biopharm, Darmstadt, Germany) and using a Sunrise™ plate reader at 450 nm (Tecan Group Ltd., Männedorf, Switzerland).

Ochratoxin A (OTA, µg/kg) was determined following the Ridascreen^®^ Ochratoxin A 30/15 procedure (R-Biopharm, Darmstadt, Germany) and using a Sunrise™ plate reader at 450 nm (Tecan Group Ltd., Männedorf, Switzerland).

### 4.3. Agrometeorologic Parameters

The agrometeorological parameters (air temperature, precipitation, and soil water reserve) in the extremely hot and dry 2023–2024 agricultural year were registered by the official stations of the National Meteorological Administration (Meteo-Romania).

The facilities hold quality accreditation and certification in accordance with ISO standards. Extreme weather conditions, including heatwaves and droughts, in Romania during September 2023 to August 2024 are fully detailed in [9,24].

In the analysis of the quality of stored wheat and derived products, agrometeorological data from May to August 2024 were used (Figure 1), as they were significantly correlated with the quality of harvested wheat [9]. In the pedoclimatic conditions of Romania, the phenological stages of common wheat are earing in May, anthesis at the end of May and June, grain filling in June and July, and full maturity and harvest at the end of July and August [8].

### 4.4. Statistical Analysis

Data were collected in a database and statistically evaluated with JASP Team software version 0.17.1 (University of Amsterdam, The Netherlands). Linear correlations were calculated between microbiological, mycotoxicological, physico-chemical, rheological, and sensory-colorimetric parameters in stored wheat, wholemeal flour, and bread, and the agrometeorological parameters in May–August 2024. Three thresholds were used to interpret significance: significant correlation * (*p*-value < 0.05), distinctly significant correlation ** (*p*-value < 0.01), and highly significant correlation *** (*p*-value < 0.001).

The Pearson correlation coefficient was applied based on the linear relationship between quantitative variables, their normal distribution (as determined by the Shapiro-Wilk test), and the absence of outliers. This method enabled the identification of predictive associations, forming a basis for future analyses that will incorporate spatial and temporal dimensions, including historical data.

This study presents the Pearson correlations between microbiological and mycotoxicological parameters with physico-chemical, rheological, and sensory-colorimetric parameters of stored wheat, wholemeal flour, and wholemeal bread:

(a). Physico-chemical parameters of wheat: hectoliter mass (HM, kg/hectolitre); Hagberg falling number (HFN, seconds); protein (P, % dry matter); wet gluten (WG, %); wet gluten deformation index (WGDI, mm); gluten index (GI).

(b). Physico-chemical and rheological parameters of flour: moisture (M, %); total titrable acidity (TTA, %); starch (%); maximum pressure resistance of dough (P, mbar); extensibility and curve length of dough (L, mm); P/L ratio; swelling index of dough (G, mm); power of dough (W, 10 E^−4^ J); elasticity index of dough (IE). Rheological parameters were determined with a Dough Rheology Analyzer AlveoPC, series 7165 (Chopin, Villeneuve-la-Garenne, France).

(c). Physico-chemical parameters of bread: moisture (M, %); total titrable acidity (TTA, %); volume (V, cm^3^); protein (P, % d.m.); fat (%); ash (%).

Specific equipment was utilized for each determination of (a), (b), and (c). The laboratory methods are accredited by the RENAR.

(d). Sensory–colorimetric parameters: L*—sample brightness on a scale from 0 to 100 (L* = 0, black; L* = 100, white); a*—sample color on a scale from pure green to pure red (−a, green; +a, red); and b*—sample color on a scale from pure blue to pure yellow (−b, blue; +b, yellow). Parameters were determined with a CM-5 spectrophotometer (Konica Minolta, Tokyo, Japan). The sensory-colorimetric indicators of Romanian wheat fall within the spectrum towards white, red, and yellow.

Pearson correlation coefficients are presented in Tables S1–S4 in the Supplementary Materials.

### 4.5. Geographic Distribution

The spatial distribution of microbiological and mycotoxicological parameters was visualized using Excel’s Map Chart feature, which employs the geography data types available in Microsoft 365 Business Standard, version 2508 (Microsoft, Redmond, Washington, DC, USA) (Figure 1, Figure 2, Figure 3, Figure 4, Figure 5, Figure 6, Figure 7 and Figure 8). 

Romania has 41 counties and the municipality of Bucharest, which has the status equivalent to a county, together constituting basic administrative units for territorial organization and local government. All counties are delimited in Figure 1, Figure 2, Figure 3, Figure 4, Figure 5, Figure 6, Figure 7, Figure 8 and Figure 9 and clearly named in Figure 1a–c and Figure 9. The counties from which the stored wheat samples were purchased are named and highlighted in colors in Figure 2, Figure 3, Figure 4, Figure 5, Figure 6, Figure 7, Figure 8 and Figure 9.

## Data Availability

The original contributions presented in this study are included in the article and Supplementary Materials. Further inquiries can be directed to the corresponding author.

## Supplementary Materials

The following are available online at https://www.mdpi.com/article/10.3390/toxins17100502/s1. Table S1. Pearson correlations between microbiological and mycotoxicological indicators of stored wheat, wholemeal flour, and bread, and the agrometeorological parameters in 2024; Table S2. Pearson correlations between microbiological and mycotoxicological indicators of stored wheat, wholemeal flour, and bread; Table S3. Pearson correlations between microbiological and mycotoxicological indicators and the physicochemical and rheological indicators of stored wheat, wholemeal flour, and bread; Table S4. Pearson correlations between microbiological and mycotoxicological indicators and the sensory-colorimetric indicators of stored wheat, wholemeal flour, and bread.
