# Supplementary material for: Microbiological and Mycotoxicological Quality of Stored Wheat, Wholemeal Flour and Bread: The Impact of Extreme Weather Events in Romania in the 2024 Summer
Source: Toxins (Basel). 2025 Oct 11;17(10):502. doi: 10.3390/toxins17100502 (PMC12567793; doi:10.3390/toxins17100502)
Supplement: Supplementary file 1 [file toxins-17-00502-s001.zip › toxins-3893187-supplementary.pdf]

toxins

MDPI

Supplementary Materials: Microbiological and Mycotoxicological Quality of Stored Wheat, Wholemeal Flour and Bread: The Impact of Extreme Weather Events in Romania in the 2024 Summer

Table S1. Pearson correlations between microbiological and mycotoxicological indicators of stored wheat, wholemeal flour, and bread, and the agrometeorological parameters in 2024.

Pearson Correlations (r_{xy}) between Microbiological and Mycotoxicological Indicators of Stored Wheat, Wholemeal Flour, and Bread, and the Agrometeorological Parameters in 2024													
**Product**	**Microbiological and Mycotoxicological Indicators**	**Agrometeorological Parameters in the 2024 Summer**											
		Average Air Temperature					Cumulative Precipitation					Average Soil Water Reserve	
		**May**	**June**	**July**	**August**	**May–Aug.**	**May**	**June**	**July**	**August**	**May–Aug.**	**May**	**June**
**Stored Wheat**	**Moisture**	-0.209	0.336	0.277	0.190	0.182	-0.045	-0.134	-0.616*	-0.352	-0.425	-0.106	-0.181
	**Total Fungi**	0.363	-0.136	-0.047	0.058	0.040	-0.222	0.080	0.299	-0.001	0.076	-0.100	0.034
	***Fusarium*-damaged kernel**	-0.666*	-0.693**	-0.704**	-0.736**	-0.729**	-0.281	0.315	0.158	0.500	0.294	-0.404	0.049
**Wholemeal Flour**	**Moisture**	-0.674*	-0.635*	-0.677*	-0.716**	-0.703**	-0.106	0.450	0.112	0.577*	0.420	-0.115	0.419
	**Water activity**	-0.745**	-0.676*	-0.697**	-0.737**	-0.740**	-0.135	0.462	0.190	0.608*	0.457	-0.243	0.288
	**Total Fungi**	0.231	-0.040	-0.017	-0.005	0.028	-0.16	0.563*	0.146	0.199	0.323	0.378	0.779**
	**Deoxynivalenol**	-0.816***	-0.753**	-0.822***	-0.852***	-0.843***	-0.021	0.518	0.330	0.639*	0.580*	-0.081	0.418
	**Aflatoxin B1**	-0.377	-0.684**	-0.646*	-0.637*	-0.625*	-0.286	0.206	0.474	0.654*	0.409	0.199	0.381
	**Ochratoxin A**	0.173	-0.192	-0.182	-0.156	-0.114	0.250	0.083	0.398	0.365	0.390	0.348	0.276
**Wholemeal Bread**	**Moisture**	-0.387	-0.304	-0.302	-0.326	-0.339	-0.182	0.218	0.274	0.287	0.244	-0.354	-0.161
	**Water activity**	-0.366	-0.678*	-0.694**	-0.657*	-0.641*	-0.091	0.197	0.487	0.631*	0.466	0.183	0.442
	**Total Fungi**	n.a.	n.a.	n.a.	n.a.	n.a.	n.a.	n.a.	n.a.	n.a.	n.a.	n.a.	n.a.
	**Deoxynivalenol**	-0.522	-0.536	-0.563*	-0.567*	-0.571*	0.308	0.401	0.373	0.557*	0.616*	0.520	0.491
	**Aflatoxin B1**	0.326	0.025	0.013	0.037	0.086	-0.257	0.058	-0.197	-0.077	-0.159	0.038	0.251
	**Ochratoxin A**	-0.133	-0.192	-0.244	-0.258	-0.222	0.166	0.034	0.365	0.264	0.296	-0.019	0.031

Significance (two-tailed): * *p*-value < 0.05—significant differences, ** *p*-value < 0.01—distinct significant differences, *** *p*-value < 0.001—very significant differences.

Table S2. Pearson correlations between microbiological and mycotoxicological indicators of stored wheat, wholemeal flour, and bread.

Pearson Correlations (r_{xy}) between Microbiological and Mycotoxicological Indicators of Stored Wheat, Wholemeal Flour, and Bread								
**Product**	**Microbiological and Mycotoxicological Indicators**	**Microbiological and Mycotoxicological Indicators**						
		**Moisture**	**Water activity**	**Total Fungi**	***Fusarium*-damaged kernel**	**Deoxynivalenol**	**Aflatoxin B1**	**Ochratoxin A**
**Stored Wheat**	**Moisture**	*1.000*	-	-	-	-	-	-
	**Total Fungi**	-0.743**	n.a.	*1.000*	-	-	-	-
	***Fusarium*-damaged kernel**	0.152	-	-0.221	*1.000*	-	-	-
**Wholemeal Flour**	**Moisture**	*1.000*	-	-	-	-	-	-
	**Water activity**	0.985***	*1.000*	-	-	-	-	-
	**Total Fungi**	-0.039	-0.126	*1.000*	-	-	-	-
	**Deoxynivalenol**	0.791**	0.805***	-0.207	-	*1.000*	-	-
	**Aflatoxin B1**	0.296	0.278	0.065	-	0.351	*1.000*	-
	**Ochratoxin A**	-0.095	-0.102	0.280	-	0.200	0.375	*1.000*
**Wholemeal Bread**	**Moisture (M)**	*1.000*	-	-	-	-	-	-
	**Water activity**	0.998***	*1.000*	-	-	-	-	-
	**Total Fungi**	-	n.a.	-	-	-	-	-
	**Deoxynivalenol**	0.174	0.469	-	-	*1.000*	-	-
	**Aflatoxin B1**	0.231	0.087	-	-	-0.400	*1.000*	-
	**Ochratoxin A**	0.257	0.280	-	-	0.209	-0.032	*1.000*

Significance (two-tailed): * p-value < 0.05—significant differences, ** p-value < 0.01—distinct significant differences, *** p-value < 0.001—very significant differences.

Table S3. Pearson correlations between microbiological-mycotoxicological indicators and the physico-chemical and rheological indicators of stored wheat, wholemeal flour, and bread.

Pearson Correlations (r_{xy}) between Microbiological and Mycotoxicological Indicators and the Physicochemical and Rheological Indicators of Stored Wheat, Wholemeal Flour, and Bread																					
Product	Microbiological and Mycotoxicological Indicators	Physicochemical and Rheological Indicators																			
		Stored Wheat						Wholemeal Flour								Wholemeal Bread					
		Hectoliter Mass	Moisture	Hagberg Falling Number	Protein	Wet Gluten	Gluten Index	Total Titrable Acidity	Starch	Maximum Pressure Resistance of Dough	Extensibility and Curve Length of Dough	P/L Ratio	Swelling Index of Dough	Power of Dough	Elasticity Index of Dough	Volume	Moisture	Total Titrable Acidity	Protein	Fat	Ash
Stored Wheat	**Moisture**	0.069	*1.000*	-0.199	0.122	-0.046	0.432	-	-	0.497	0.089	0.292	0.090	0.471	0.203	-	-	-	-	-	-
	**Total Fungi**	-0.051	-0.743**	-0.171	0.072	0.369	-0.042	-	-	-0.204	0.326	-0.355	0.319	-0.014	0.061	-	-	-	-	-	-
	***Fusarium*-damaged kernel**	-0.629*	0.152	0.089	-0.350	-0.642*	-0.437	-	-	-0.462	-0.116	-0.207	-0.120	-0.427	0.068	-	-	-	-	-	-
Wholemeal Flour	**Moisture**	-	*1.000*	-	-0.257	-0.445	-0.243	0.752***	0.920***	-0.384	0.166	-0.340	0.172	-0.057	-	-	-	-	-	-	-
	**Water Activity**	-	0.985***	-	-0.171	-0.358	-0.227	0.719***	0.861***	-0.346	0.089	-0.284	0.096	-0.124	-	-	-	-	-	-	-
	**Total Fungi**	-	-0.039	-	-0.316	-0.328	-0.400	0.463	0.220	0.152	0.328	-0.084	0.335	0.432	-	-	-	-	-	-	-
	**Deoxynivalenol**	-	0.791**	-	-0.093	-0.255	-0.250	0.125	0.107	-0.724**	0.330	-0.622*	0.336	-0.175	-	-	-	-	-	-	-
	**Aflatoxin B1**	-	0.296	-	-0.508	-0.426	-0.609*	0.033	0.182	-0.281	0.220	-0.261	0.203	0.055	-	-	-	-	-	-	-
	**Ochratoxin A**	-	-0.095	-	-0.443	-0.491	-0.074	0.278	0.147	0.160	-0.223	0.209	-0.214	-0.107	-	-	-	-	-	-	-
Wholemeal Bread	**Moisture**	-	-	-	-	-	-	-	-	-	-	-	-	-	-	0.465	*1.000*	0.874***	0.894***	0.840***	0.910***
	**Water Activity**	-	-	-	-	-	-	-	-	-	-	-	-	-	-	0.458	0.998***	0.875***	0.887***	0.837***	0.901***
	**Total Fungi**	-	-	-	-	-	-	-	-	-	-	-	-	-	-	-	-	-	-	-	-
	**Deoxynivalenol**	-	-	-	-	-	-	-	-	-	-	-	-	-	-	0.235	0.174	0.355	0.268	0.161	0.218
	**Aflatoxin B1**	-	-	-	-	-	-	-	-	-	-	-	-	-	-	0.312	0.231	0.205	0.195	0.473*	0.239
	**Ochratoxin A**	-	-	-	-	-	-	-	-	-	-	-	-	-	-	0.453	0.257	0.314	0.306	0.299	0.384

Significance (two-tailed): * *p*-value < 0.05—significant differences, ** *p*-value < 0.01—distinct significant differences, *** *p*-value < 0.001—very significant differences.

Table S4. Pearson correlations between microbiological and mycotoxicological indicators and the sensory-colorimetric indicators of stored wheat, wholemeal flour, and bread.

Pearson Correlations (r_{xy}) between Microbiological and Mycotoxicological Indicators and the Sensory-Colorimetric Indicators of Stored Wheat, Wholemeal Flour, and Bread				
**Microbiological and Mycotoxicological Indicators**		**Sensory-Colorimetric Indicators**		
		**L*–whiteness**	**a*–redness**	**y*–yellowness**
**Stored Wheat**	**Moisture**	n.a.	n.a.	n.a.
	**Total Fungi**	n.a.	n.a.	n.a.
	***Fusarium*-damaged kernel**	n.a.	n.a.	n.a.
**Wholemeal Flour**	**Moisture**	0.592*	-0.624*	-0.665*
	**Water activity**	0.521	-0.580*	-0.618*
	**Total Fungi**	0.480	-0.317	0.004
	**Deoxynivalenol**	0.552	-0.662*	-0.493
	**Aflatoxin B1**	0.440	-0.529	-0.369
	**Ochratoxin A**	0.349	-0.355	-0.373
**Wholemeal Bread**	**Moisture**	-0.052	0.046	-0.156
	**Water activity**	-0.438	-0.127	-0.380
	**Total Fungi**	n.a.	n.a.	n.a.
	**Deoxynivalenol**	-0.450	0.236	0.049
	**Aflatoxin B1**	-0.276	-0.410	-0.418
	**Ochratoxin A**	-0.110	0.302	-0.081

Significance (two-tailed): * *p*-value < 0.05—significant differences, ** *p*-value < 0.01—distinct significant differences, *** *p*-value < 0.001—very significant differences. Stored wheat, n.a.—analyses were performed on wholemeal flour. Wholemeal bread, n.a.—total fungi <10 ufc/g.
